# Prey Responses to Predator Chemical Cues: Disentangling the Importance of the Number and Biomass of Prey Consumed

**DOI:** 10.1371/journal.pone.0047495

**Published:** 2012-10-17

**Authors:** Michael W. McCoy, Justin C. Touchon, Tobias Landberg, Karen M. Warkentin, James R. Vonesh

**Affiliations:** 1 Department of Biology, East Carolina University, Greenville, North Carolina, United States of America; 2 Department of Biology, Virginia Commonwealth University, Richmond, Virginia, United States of America; 3 Smithsonian Tropical Research Institute, Balboa, República de Panamá; 4 Department of Biology, Boston University, Boston, Massachusetts, United States of America; California State University Fullerton, United States of America

## Abstract

To effectively balance investment in predator defenses versus other traits, organisms must accurately assess predation risk. Chemical cues caused by predation events are indicators of risk for prey in a wide variety of systems, but the relationship between how prey perceive risk in relation to the amount of prey consumed by predators is poorly understood. While per capita predation rate is often used as the metric of relative risk, studies aimed at quantifying predator-induced defenses commonly control biomass of prey consumed as the metric of risk. However, biomass consumed can change by altering either the number or size of prey consumed. In this study we determine whether phenotypic plasticity to predator chemical cues depends upon prey biomass consumed, prey number consumed, or both. We examine the growth response of red-eyed treefrog tadpoles (*Agalychnis callidryas*) to cues from a larval dragonfly (*Anax amazili*). Biomass consumed was manipulated by either increasing the number of prey while holding individual prey size constant, or by holding the number of prey constant and varying individual prey size. We address two questions. (i) Do prey reduce growth rate in response to chemical cues in a dose dependent manner? (ii) Does the magnitude of the response depend on whether prey consumption increases via number or size of prey? We find that the phenotypic response of prey is an asymptotic function of prey biomass consumed. However, the asymptotic response is higher when more prey are consumed. Our findings have important implications for evaluating past studies and how future experiments should be designed. A stronger response to predation cues generated by more individual prey deaths is consistent with models that predict prey sensitivity to per capita risk, providing a more direct link between empirical and theoretical studies which are often focused on changes in population sizes not individual biomass.

## Introduction

The nonlethal effects of predators on prey phenotype and performance can affect prey fitness [1], [2], the outcome of predator-prey interactions, and influence the long term properties of communities [3], [4]. Over the past two decades a growing body of literature has revealed the importance of predator-induced changes in prey traits and their influence on food web dynamics in a wide array of organisms and ecological systems [5], [6]–[10]. However, the magnitude of predator-induced phenotypic change and direction of the effect of trait-mediated interactions have been inconsistent among studies and systems [5], [10]. Indeed, a number of studies have highlighted the context dependence of non-lethal effects of predators on prey phenotype [11], [12]–[18] and indirect interactions with other members of the community [4], [10]. Understanding the mechanisms that lead to different degrees of phenotypic response to predators is an important step towards understanding the context dependence of trait-mediated interactions and for synthesizing and generalizing patterns within and across study organisms and systems.

To effectively balance investment in morphological and behavioral defenses while maintaining investment in other traits (e.g. growth and development), organisms must assimilate information from the environment that accurately reflects both the presence of predators and the relative risk that they impose [12], [17], [19]–[21]. Organisms often use visual, chemical, auditory and vibrational cues from predators, conspecifics, and other species to identify an elevated risk of predation [22], [23]–[29]. Chemical cues are an important source of information about environmental quality in both aquatic [23], [30] and terrestrial systems [31], [32] and organisms are known to be able to detect and distinguish among different species of predators [27], [33]–[36], different predator diets [30], [37], predator density [17], [18] and prey density [12], [18]. Indeed, consistent with theory [19], [38] there is a growing evidence that organisms use chemical cues to not only assess the presence of predators, but also to determine the magnitude of risk posed by those predators [12], [13], [17], [18]. How organisms gauge the magnitude of risk posed by predators is, however, still not well understood.

Studies aimed at quantifying predator-induced defenses in aquatic systems commonly attempt to control for variation in chemical cue concentration (i.e., perceived predation risk) by feeding caged predators a fixed biomass of prey [17], [20], [35]. However, per capita predation rate, the *number* (not biomass) of prey consumed per predator per time, is often used as the metric of relative risk [39] and is typically modeled as a function of predator attack rate, handling time and prey density. Because the biomass of prey consumed can increase in two manners–by increasing either the number of individuals or the size of individuals consumed–it is unclear if the biomass of prey eaten provides information about per capita risk. Indeed, the number of prey eaten should be a more relevant indicator of per capita risk. If that is the case, maintaining a constant biomass to generate chemical cues of predation risk, at the expense of a decreasing (as prey grow) or variable number of prey consumed, may lead to unintended systematic variation in perceived risk that is compounded through time.

The objectives of this study were to determine the effects on prey phenotype of controlling for biomass via the size versus the number of prey consumed by predators. We test the predictions that prey respond to chemical cues of predation in a dose dependent manner, and that–as a more reliable indicator of risk–prey respond more strongly to cues generated on a per capita rather than a per biomass basis.

## Materials and Methods

This research was conducted at the Smithsonian Tropical Research Institute, Gamboa, Panama in summer 2011. All necessary permits were obtained from Autoridad Nacional del Ambiente de Panamá to conduct this research in Panama (Permiso No. SC/A-13-11) and from IACUC protocol approval (2011-0616-2014-04). Our general design entailed manipulating cues of a common predator (larval dragonfly, *Anax amazili*) by varying the biomass of prey consumed (*Agalychnis callidryas* tadpoles) in one of two ways and quantifying the short-term growth response of the prey (duration: 8 days). Prey biomass consumed was manipulated by either increasing the number of individuals (holding individual prey size constant), or by holding the number of prey individuals constant and varying individual prey size. Specifically, our experiment had 3 treatments: 1) No predator, 2) variable number of single sized prey (1, 4, 10, 20 hatchlings*d^−1^) or 3) a single prey d^−1^ of variable sizes [size class (mean individual mass g ±1 sd): hatchling (0.021±0.009), small (0.08±0.019), medium (0.18±0.025) or large (0.37±0.04)]. We provided the predators varying amounts of prey to ensure we generated variation in cue strength, but because predators did not always eat all prey provided (see results) we quantified the actual biomass of prey consumed by predators and used these values as a continuous predictor of phenotypic response in our statistical analyses. The average prey biomass fed to predators in treatments with single and increasing numbers of prey spanned a similar range (single: 0.021–0.37 g, multiple: 0.021–0.42 g). Each cue treatment level was replicated 9 times in 72 400 L mesocosms (0.7 m diameter base, 0.9 m diameter mouth×0.8 m high, with screened drain holes at 0.75 m height) arrayed in a partially shaded field adjacent to forest. Mesocosms were filled with a mix of rain water and aged tap water 1–2 days prior to the start of the experiment and were provided with sufficient leaf litter to cover the bottom (∼50 large leaves) and 2.0 g of Sera micron ® powdered fish food. Each tank received 10 focal tadpoles. Focal tadpoles were obtained from 24 clutches laid on 24 June at Experimental pond in Gamboa, Panama. These clutches were maintained in the lab until we induced hatching by submerging and manually stimulating embryos at 6 days post-oviposition. Hatchlings (>1000 total) were pooled then haphazardly allocated into groups of 10 that were randomly assigned to replicates. All focal tadpoles were digitally photographed in dorsal view in a shallow white tray containing a ruler to quantify initial (1 July) and final (9 July) total length using the program ImageJ [40].

All predator treatments contained two individually caged late instar *A. amazili* nymphs [mean length (mm ±1 sd) 29.44±3.84, N = 149]. Dragonflies were field collected by dip-net from Quarry Pond in Gamboa on 28–30 June, fed 1 hatchling tadpole, then starved until haphazardly assigned to treatments on 1 July. Cages consisted of 475 ml plastic cups with a small hole punched in the bottom and covered with screen (tulle) held on by elastic. Cups were hung upside down from cross wires at the top of each mesocosm by a clothes pin attached to the cup with screens 3–5 cm below the waters’ surface and holes in the air.

Predators were fed daily. Treatments receiving the smallest size class of prey were provided 6–7 d post-oviposition hatchlings from clutches collected from Experimental Pond, first from same cohort as focal tadpoles and then from clutches laid in subsequent days. Tadpoles for treatments receiving larger prey were collected from Experimental Pond each day and sorted visually into respective size classes. For treatments receiving only 1 prey d^−1^, we alternated which dragonfly was fed. Concurrent studies of ours with *A. amazili* and *A. callidryas* have demonstrated that unfed predators have no effect on the growth of hatchling tadpoles and that predator number has no effect independent of prey consumed (J. R. Vonesh, K. M. Warkentin unpublished data). Any tadpoles remaining in predator cages from the previous day were recorded and removed, and partially consumed tadpoles were weighed, to allow us to estimate actual prey biomass d^−1^ versus simply biomass provided. Predators that died or metamorphosed were replaced (N = 20). At the start of the experiment dragonflies were digitally photographed in dorsal view in a shallow white tray containing a ruler for total length measurement. Replacement dragonflies and feeder tadpoles were also photographed for length measurements.

Change in tadpole total length (i.e. growth) was used as the phenotypic response variable in our analysis. We focused only on total length because we have previously tested for morphological plasticity of 8 different morphological traits commonly examined in tadpoles in response to the chemical cues of predation risk from 5 different species of predators (including *A. amazili*), and found total length to be the only trait in which a phenotypic response is detectable (Vonesh, Touchon, Warkentin and McCoy unpublished, and [41]). Moreover, total length is strongly correlated with body mass (R^2^ = 0.99, [41]) and therefore is a good metric of an overall size response in this species.

To determine if there was a significant difference between the growth rates of tadpoles exposed to chemical cues generated from consumption of one versus multiple feeder tadpoles we first tested for differences in the initial sizes (to insure there were no difference in initial conditions) of tadpoles in our eight feeding regimes using analysis of variance (none were detected–see results). To test for differences in the magnitude of phenotypic responses induced by predators fed one versus more than one prey we calculated the percent difference in the growth of tadpoles in the two predator treatment groups (predator fed 1 prey vs >1 prey) from the average growth of tadpoles in the control (no predator) treatment. We then fit Michaelis-Menton functions to these data using maximum likelihood to estimate model parameters. Inferences about treatment effects on model parameters were based on Likelihood Ratio Tests (LRT). All statistical analyses were performed in the R statistical programming environment [42]–maximum likelihood estimation was performed using the mle2 function in package bbmle [43]. For statistical analyses, model assumptions and model fits were evaluated visually via examination of residuals and quantile plots (ANOVA) or likelihood profile plots (maximum likelihood fits).

## Results

There were no differences in the initial size of tadpoles among treatments (TL (mm) ±1 sd: 13.46±0.58; F_7,82_ = 1.47, p = 0.19) and all focal tadpoles survived. Consumption of cue tadpoles by predators was high but varied (mean number of prey consumed treatment^−1^ d^−1^±1 sd: 1-Hatchling–1±0; 4-Hatchlings–3.89±0.13; 10-Hatchlings–8.56±1.23; 20-Hatchlings–14.1±1.86; 1-Small–0.97±0.06; 1-Medium–0.99±0.04; 1-Large–0.88±0.13). Thus, the range of prey biomass consumed in predator cue treatments with single and increasing numbers of prey were nearly identical (single: 0.021–0.37 g, multiple: 0.021–0.38 g). Focal tadpoles were dramatically smaller with predator cues and tadpole growth responses were asymptotically dependent on biomass consumed (Mean final TL (mm) ±1 sd: No Predator–29.94±2.66; 1-Hatchling–25.71±1.71; 4-Hatchlings–21.31±2.25; 10-Hatchlings–19.9±1.443; 20-Hatchlings–19.07±1.18; 1-Small–21.60±1.43; 1-Medium–21.04±0.69; 1-Large–21.04±1.63).

**Figure 1:**
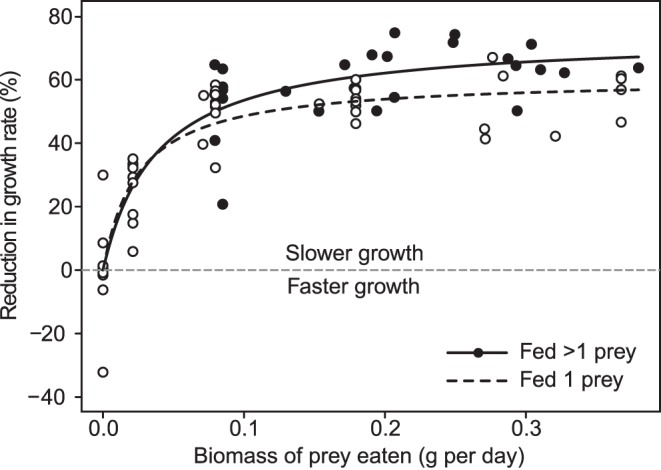
Growth suppression of red-eyed treefrog (*Agalychnis callidryas*) tadpoles through 8 days of exposure to indirect chemical cues of predation from dragonfly nymph predators (*Anax amazili*) fed different biomasses and numbers of *A. callidryas* prey. The y-axis depicts percent growth reduction, which reflects differences in the total lengths of tadpoles from the beginning to the end of the experiment relative to the mean growth of tadpoles in the predator-free controls. Thus larger values indicate greater reductions in growth. Growth suppression was dose-dependent and its asymptotic magnitude was greater when predators ate multiple prey than when they ate single prey of equivalent biomass.

Increasing the biomass of prey consumed by increasing prey size and by increasing prey number did not affect tadpole growth in the same way. Tadpoles reduced their growth as prey biomass increased, but did so more strongly when prey number also increased. Specifically, there was a significant effect of predator feeding treatment on the asymptotic phenotypic effect size (χ^2^ = 5.32, p = 0.02), but not on the rate of increase in the phenotypic effect size (χ^2^ = 1.47, p = 0.23). The asymptotic maximum effect size was 13% larger when tadpoles were reared with predators fed multiple small prey than when reared with predators fed 1 large tadpole (Figure 1).

## Discussion

Adaptive phenotypic plasticity allows organisms to adjust phenotype expression to match environmental conditions, and these changes can be critical for survival [43]–[45]. Defenses induced by natural enemies are a common form of adaptive plasticity whose effects can propagate through food webs to influence population and community dynamics via changes in prey survival and performance (e.g. growth) [3], [4], [46]. Several theoretical studies have suggested that organisms should respond to fine scale changes in the risk environment and express graded phenotypes that balance the risk of mortality with costs often associated with induced phenotypes [19], [21], [38], [45].

The most novel and potentially important finding of our study was that increasing both the number and biomass of prey consumed by predators induced a stronger phenotypic change than did increasing the biomass of prey consumed alone. After only 8 days tadpoles were 13% smaller when predators ate multiple small prey than when predators ate a single large prey each day, which translated into more than 2 mm difference in total lengths. Given that it typically takes 35 days or longer for this species to metamorphose [47], the compounding effects of this difference could lead to substantial differences in survival and time to and size at metamorphosis [34], [41], [47]–[49]. In fact, McCoy et al. [41] demonstrated that with a less lethal dragonfly naiad predator (*Pantala flavescens*), a 2 mm difference in size, like we find here, produces more than a 5% difference in daily per capita survival. Thus, the effects of different means of administering chemical cues of predation has important implications for interpreting growth, survival and demographic effects observed in risk assessment studies. Ecologist need to carefully consider which methods were used to control chemical cue concentrations when evaluating existing studies of risk assessment and what method should be used when conducting future experiments. Indeed, we are not aware of any other studies that have controlled for both the biomass and number of prey provided to predators to generate chemical cues of predation. Commonly, investigators attempt to minimize variation in chemical cues during an experiment by feeding predators a constant biomass or constant number of prey. These approaches assume either that the chemical cue used to assess predation risk is determined by the biomass of prey eaten, or that the amount of cue produced is independent of prey size, respectively. However, our study shows that increasing the biomass of prey eaten while holding number constant, and increasing both the biomass and number of prey eaten, do not produce equivalent responses. Although we found a significant decrease in the growth of tadpoles as the biomass of prey consumed by predators increased, the magnitude of this phenotypic response was significantly greater when there was a simultaneous increase in the number of prey consumed (Figure 1). Thus, many small prey are not equivalent to few large prey. An experiment designed to compare the effects of low and high cue concentrations that controls for biomass, but not the number of prey used to generate cue, could produce misleading results. For example, if “high cue” treatments consist of large tadpoles and “low cue” treatments consist of small tadpoles, one could observe lower or opposite than expected differences in prey responses between the two treatments. Similarly, unintended systematic variation in prey phenotypic responses could be generated in longer-term experiments if average feeder tadpole size increases through time and only biomass of prey consumed is held constant. The effects of controlling only the biomass of prey consumed by predators when generating predation cues, without regard for the mechanism by which biomass manipulated, could also have implications for studies designed to assess relative sensitivity at different points during ontogeny. For instance, controlling for only the biomass [50] or the number [51] of prey consumed by predators could lead to either lower or higher than intended cues of predation for focal prey. These effects could be erroneously perceived as changes in responsiveness of prey to predator cues at different times in ontogeny.

One hypothesis for the discord between the between the effect of increasing biomass consumed via numerical or size based mechanisms is that the chemical cue that indicates predation threat (kairomone) is located in the skin or other external tissues of the tadpoles [52]. If this is the case, the surface area for a given biomass increases as the number of prey increases. Alternatively, the alarm cue may be a metabolite that scales with individual metabolic rate in which case we might also expect the chemical cue released by an individual to scale with body size in a similar way as does mass-specific metabolic rate [53], [54]. In either case, small individuals would be expected to produce proportionally more cue, for their mass, than do large individuals. Another important way that adding more individuals at a constant biomass could produce a stronger response is that the time course over which prey are eaten could be greater and so the time course of cue release may differ. For example, consumption of one large prey results in one cue release event, whereas consuming 10 tadpoles with the same total biomass presumably results in 10 cue release events which likely occur over a longer time frame resulting in a more steady release of cue as prey are consumed in sequence. Finally, the asymptotic effects of increasing prey consumed by the predators may be the result of different mechanisms in the two scenarios. For example, if the amount of cue that can be released per prey were asymptotic with prey size then the asymptotic effects would be driven by the amount of cue released in the single prey scenario, and by the ability of the prey to respond in the multiple prey scenario. Understanding the localized source, identity, rate of release and persistence, as well as the scaling of alarm cues with prey size will have important implications for understanding risk assessment, and should be the focus of future research.

Although the identity of the specific chemicals and the persistence of chemical cues used to assess predation threat are largely unknown across systems, there are several steps that experimentalists can take to minimize the potential confounding effects of changing cue concentrations in experiments. First, whenever possible, experiments should control for both the biomass of prey as well as the number of prey eaten by predators (or that are otherwise manipulated to generate cues of mortality risk). When such controls are not possible, investigators should, at a minimum, report both the biomass and number of prey used to generate cue over the course of the experiment. A better appreciation for how indirect cues of predation risk are produced and used to assess risk will inform quantitative attempts to synthesize the literature and generalize observations across studies and systems via meta-analysis or other synthetic approaches. For example, if the amount of cue released scales predictably with body size then knowing the number and biomass of prey used enables quantitative comparisons of phenotypic responses as a function of cue concentration across studies. Currently, much variation among studies in both the magnitude of phenotypic responses observed as well as in the fitness consequences of those responses could be driven by differences in actual and perceived risk for prey. Reconciling this variation will have important implications for both empirical and theoretical research. Most predator-prey theory predicts per capita risk to prey to be a function of number of prey consumed, while most studies of predator-induced defenses have treated chemical cues of predation risk either as being present/absent or as a function of biomass of prey consumed by the predators. Thus, our finding that the magnitude of phenotypic response to predation increased more with more predation events may provide a critical link from data on the magnitude of phenotypic responses and their fitness consequences to models designed to predict the long-term population dynamic consequences of induced defenses.
